# Genus hologram antenna for MIMO applications

**DOI:** 10.1038/s41598-026-50229-3

**Published:** 2026-05-07

**Authors:** Nermeen A. Eltresy, Hend A. Malhat, Saber Zainud Deen

**Affiliations:** 1 Microstrip Department, Electronics Research Institute, Cairo, 12622 Egypt; 2 Faculty of Electronic Engineering, Menoufia University, Menoufia, Egypt

**Keywords:** Engineering, Physics

## Abstract

A novel high-gain Genus Hologram Antenna (GHA) based on surface impedance modulation using a periodic array of hexagonal patches is proposed for MIMO applications. The antenna features a simple, low-profile, low-cost structure that eliminates the need for complex feeding networks while achieving frequency-dependent beam scanning, multi-beam generation, and high radiation efficiency. The proposed GHA demonstrates wide-angle beam scanning from 30° to 64° over the frequency band of 13–17 GHz, with a peak gain of 20.6 dBi and radiation efficiency of 87%. By controlling the lattice periodicity of the hexagonal patches, dual-beam radiation at (30°, 40°) and (60°, − 60°), as well as four-beam operation at (120°, 60°, − 60°, − 120°) with a gain of 15.2 dBi at 16 GHz, are successfully realized. For MIMO applications, two GHA elements are integrated with an extremely compact edge-to-edge spacing of only 4.7 mm (0.25λ₀ at 16 GHz). By strategically inserting parasitic elements between the radiating units, the mutual coupling is significantly reduced from − 10 dB to below − 20 dB across the entire operating band, while maintaining excellent diversity performance (ECC < 0.003 and DG ≈ 10). Additionally, the conformal performance of the GHA is investigated on curved surfaces in both transverse and longitudinal directions, confirming its suitability for integration on platforms such as missiles, vehicles, aircraft, and trains. The proposed Genus Hologram Antenna offers a compelling combination of high gain, wide beam-scanning range, multi-beam capability, excellent MIMO isolation, and conformality, making it a strong candidate for modern 5G/6G wireless systems and compact high-performance platforms.

## Introduction

 The rapid progress of high data rates wireless transmission technology has driven a strong demand for compact, high-performance Multiple-Input-Multiple- Output (MIMO) antennas capable of delivering high channel capacity, improved spectral efficiency, and reliable link quality without increasing bandwidth or transmit power. The utilization of MIMO antenna enhances data throughput and expands link coverage without compromising on additional bandwidth or heightened transmit power^1^. MIMO antennas have advantages of enhancing data transfer speed and extending coverage without the need for extra frequency resources, amplified transmission capability, diverse spatial and pattern characteristics, and reduced signal interruptions^2^. Multiple antennas in the MIMO system transmit and receive signals simultaneously, functioning as either transmitters or receivers based on their intended use. An array of antennas on a common substrate and operating on the same frequency is classified as a MIMO antenna. To make MIMO antenna easy for integration with monolithic integrated circuits (ICs), it should have a common ground^3^. Nevertheless, there are MIMO antennas that are not placed on the same ground plane. However, this is not advisable to enhance integration efficiency and maintain consistent voltage levels. Different MIMO antennas were introduced in^4–6^. The compactness in the MIMO antennas design on the same substrate is essential to reduce the form factor. However, more compactness increases the mutual coupling effect between the antenna elements due to the surface wave and the space wave when the spacing between MIMO antennas is less than half of the free-space wavelength^3^. Consequently, this reduces the MIMO antenna performance because of power losses in the mutual coupling. As a result of that isolation technique correlated with MIMO antenna design is essential. Different techniques of mutual coupling reduction between MIMO antenna elements were presented in^3^.

Metamaterial has the ability to enhance the manipulation of the macroscopic physical field, this leads to achieve some unique electromagnetic characteristics, such as negative refraction index^7^, negative permeability^7^, negative permittivity^8^, etc. Metamaterial was introduced and investigated^9^. The three basic properties of metamaterial are (i) artificial composite material with new physical characteristics, which are not achieved by natural materials; (ii) composite of elements with sub wavelength, usually between λ/4 and λ/10, ii) materials which properties are specified by their artificial structures not by intrinsic properties of their constituent materials. Metasurface structures are commonly used in antennas because of their superior abilities of manipulating the amplitude, phase, polarization states and the propagation modes of electromagnetic wave^10^. Initially, the metasurface is employed as an electromagnetic band gap (EBG) structure to eliminate the propagation of surface waves and minimize back lobes in antenna design^11^. Subsequently, the metasurface is increasingly utilized as the reflective surface for antennas, which can significantly minimize the gap between the antenna and the reflective surface, resulting in a low profile^12^, owing to its in-phase reflection properties similar to a Perfect Magnetic Conductor (PMC). According to the mirror image principle, the wave reflected from the metasurface and the radiation wave emitted by the antenna can be superimposed in phase, thereby enhancing the gain and impedance characteristics of the antenna. In 2014, Liu et al.^13,14^ were the first to utilize the mushroom-shaped metasurface as elements for antenna radiation, successfully obtaining a wide bandwidth and significant gain in a low-profile design via slot coupling. Subsequently, a significant number of novel antennas utilizing metasurface as radiation elements are implemented, which include polarization rotation antennas^15,16^, frequency reconfigurable antennas^17^, and miniaturized antennas^18^.

In MIMO system, the base station (BS) is equipped with a large antenna array for increasing the spectral efficiency (SE). Therefore, employing a sufficiently large number of antenna arrays, the throughput can be enhanced in a way that scales with the number of base station antennas^19^. In^20,21^ the theoretical advantages of SE for MIMO systems were introduced. Nevertheless, investigating a massive MIMO BS equipped with a normal antenna array, capable of realizing these benefits, is still a very challenging task. This due to the difficulties in large-scale antenna arrays such as high cost, increased power consumption, and constrained physical size and shape^19^. Various signal processing techniques have been explored to address these challenges. Such as analog combining to reduce the size and cost of the system^22^; implementing lower resolution quantization and/or antenna selection to mitigate the power consumption^23^; and applying efficient power amplifiers operating at low peak-to-average-power ratio^24^. However, all these techniques adopt a fixed optimal antenna array, and seek to tackle the difficulties which arise from this antenna array architecture from a signal processing perspective. To avoid this dynamic metasurface antennas (DMAs) have been introduced that can realize planar, compact, low cost, and spectral efficient massive MIMO BSs. Unlike standard analog combining with conventional antenna arrays, DMAs implement adjustable compression without requiring additional hardware. DMAs are implemented by electrical tuning of the physical properties of metamaterial antennas. DMAs Apply signal processing techniques such as beamforming, analog combining, compression, and antenna selection, without additional hardware. The electrical tuning of the physical properties DMAs is achieved by using solid-state switchable components into each metamaterial element and managing them independently. The DMAs are simple, fast, planar, and low-power systems for microwave imaging, radar systems, and satellite communications^19^. With the compact size and low power consumption of DMAs, they are a promising solution for improving Energy Efficiency (EE)^25^. The movable antenna^26^ enables changing location of wireless channel at the transmitter/receiver through changing the three-dimensional positions and/or 3D rotations of antennas. This leads to enhance the performance of wireless networks and reduces cost without the requirement to deploy additional antennas. However, the channel estimation for MIMO systems that use movable antenna is challenge. In^26^ a high channel estimation accuracy is obtained with low pilot training overhead, by applying a tensor decomposition-based method for estimating the parameters of multi-path channel components, as well as their azimuth and elevation angles, also complex gain coefficients, thereby restructuring the wireless channel between any pair of Tx and Rx movable antenna positions in the Tx and Rx regions.

Structure that can radiate focused beams in a specific direction by utilizing surface waves are hologram antennas (HAs)^27,28^. Surface waves launcher and interference surface together construct the hologram patterns with focused radiation beams in a specific direction. HAs have advantages of high gain, narrow beam width, low profile, and ability to achieve beamforming and frequency scanning with multiple polarization without using complex feeding network^29,30^. HAs are essential in different applications such as near-field electromagnetic wave signal transmissions, imaging, beam shaping, and reducing the radar cross section^31–36^. Not only in microwave applications but also, HAs are used in optical applications, such as holographic imaging 4and optical data storage^37^. Modulating the propagation constant of the surface waves along the metasurface to achieve the required radiation pattern is the working principle of HAs. For steering the radiated beam from a source to specific direction, the propagation characteristics of surface waves are utilized. The surface impedance modulation procedure is applied on Artificial Impedance Surfaces (AIS) by varying the size of the AIS elements according to a predetermined phase distribution^38^. One dimension (1D) and two dimension (2D) hologram antennas were presented for manipulating the electromagnetic surface wave propagation^36,38–40^. The 1D hologram antennas have a smaller number of unit cells than the traditional 2D hologram antennas that have large number of metasurface with diverse sizes. Consequently, 1D HAs reduces the computational complexity in surface impedance calculation and reduces the antenna size than the 2D HAs^38^. In^41^ space–time modulation for holographic antennas was introduced which enables dynamic beam shaping and frequency conversion to store and reconstruct full electromagnetic wave information (amplitude and phase). A transparent metasurface antenna for vehicle integration was presented in^42^. The antenna achieved frequency-controlled beam scanning that could be practical for 6G smart surfaces and IoT device. Holographic leaky wave antenna combined holography and leaky-wave antenna was introduced in^43^, the antenna produced omnidirectional conical radiation pattern. Circularly polarized holographic metasurface with compact, low-profile structure, gain enhancement was presented in^44^ to generate circular polarization using holographic slots. In^45^ a 24 GHz holographic metasurface antenna achieved wide-angle beam steering correlated with low sidelobes.

A genus surface antenna is a specific kind of antenna that makes use of a metasurface, which is a specially designed two-dimensional surface made up of subwavelength resonating elements known as meta-atoms or unit cells^46^. These resonating elements are designed to control how electromagnetic waves are reflected or transmitted enabling genus surface antennas to achieve functionalities such as beamforming, focusing, and polarization control. The research field of surface antennas within the genus is advancing quickly, as researchers continue to investigate new implementations in telecommunications, aerospace, and healthcare^47,48^.

In this paper, hologram antennas based on hexagonal patch periodic array is presented with studying the radiation characteristics of antenna at different frequencies. The hexagonal patch periodic array is the metamaterial section of the antenna which is fed with tapered microstrip line. The analytical approach for calculating the input impedance of the unit cell is investigated. One radiation beam, dual beams, and four beams at different radiation directions are investigated using the proposed HA. The antenna is applied in MIMO application with reduction of mutual coupling between MIMO antenna elements. The introduced antenna is studied in conformal cases of both longitudinal and transverse directions. Which is different from metamaterial-based structures, antennas proposed in^11–18^.

## Implementation of AIS

**Fig. 1:**
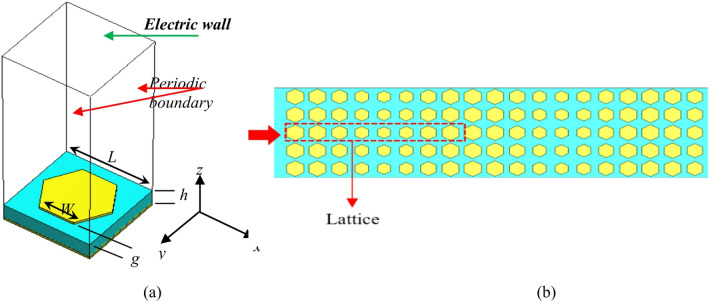
(a) Geometry of metasurface unit-cell, (b) The periodic arrangement of hexagonal metal lattice.

**Fig. 2:**
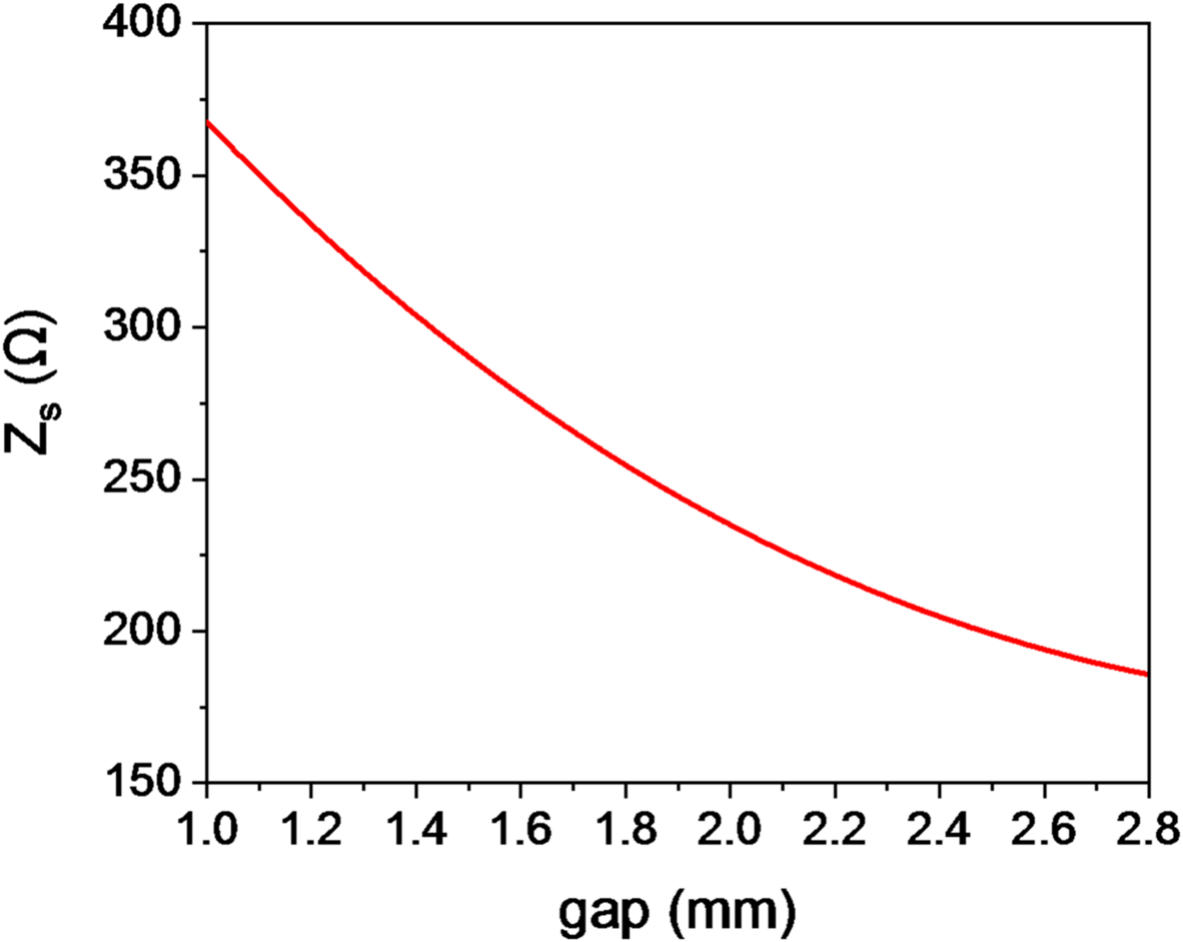
The unit cell surface impedance variation versus gap length at 16 GHz.

The unit cell consists of hexagonal patch^28^ with arm length, *W*, printed on 1.524 mm thick square grounded Rogers 4350B substrate with side length of *L*, ${\varepsilon _r}=3.5$, and *tanδ* = 0.0027. The structure of Artificial Impedance Surface (AIS) is shown in Fig. 1(a). The characteristics for surface impedance of hexagonal metal lattice as a function of geometry is obtained by simulating single unit cell as shown in Fig. 1(b). The hologram surface impedance, *Z*_*s*_, can be adjusted by changing the dimensions of the hexagonal patches, while the lattice dimension is kept constant. *Z*_*s*_ distribution is calculated using the phase distribution. The unit cell is the smallest repeating element in the proposed hologram antenna surface. It contains of metallic pattern (patch), substrate, and ground plane. The electromagnetic response of unit cell defines the whole surface. The hologram element unit cell analysis is performed using Computer Simulation Technology (CST)^49^ eigen mode solver instead of simulating a large metasurface. This reduces computational cost, allows parametric sweeps, and helps extract surface impedance (*Z*_*s*_). The unit cell is bounded by periodic boundary from XZ, YZ plans, and bounded by electric wall from XY plan as shown Fig. 1(a). The phase distribution is obtained from CST then *Z*_*s*_ distribution is calculated using the obtained phase distribution. Equation (1) provides the relation between the phase constant and the surface impedance of the hologram surface^28^.

1 $${z_s}= - \frac{{{\eta _o}{k_x}}}{{{k_o}}}=j{\eta _o}\sqrt {{{\left( {{k_z}/{k_o}} \right)}^2} - 1}$$

where${\mathrm{~}}{k_z}{\mathrm{~}}and{\mathrm{~}}{k_o}$ are the phase number in z-axis and free space, respectively. TM surface waves propagate along the + Z direction within the unit-cell, while the presence of the ground plane ensures that there are no field changes in the y-direction^28^. Figure 2 shows the *Z*_*s*_ variation with gap length at 16 GHz. A curve fitting function is applied to describe the relationship between surface impedance *Z*_*s*_ and gap *g* with mean square error (MSE) of 3 × 10^− 12^ error for the polynomial.

2 $${Z_s}= - 28.028{\mathrm{~}}{g^4}+246.38{\mathrm{~}}{g^3} - 756.93{\mathrm{~}}{g^2}+868.59{\mathrm{~}}g+3.0485$$

## Hologram antenna design

Surface wave launchers and the designed AIS are applied to build the hologram antenna. A Periodic array of small-size patches printed on a grounded dielectric substrate is formed by the AIS. The surface wave launcher generates a surface wave that is gradually converted into leaky waves based on Floquet theorem^50^. The Floquet waves propagate in periodic structures can be described by Floquet theory. For a surface with period *p*, the tangential wavenumbers of Floquet modes are *β*_*n*_ *= β*_*o*_*+(2nπ/p).* where *n* is the Floquet mode index (*n = 0*,* ± 1*,* ±2*,….). Each mode corresponds to a different radiation direction, and only modes satisfying *β*_*n*_ *< K*_*o*_ can radiate (become leaky). In the holographic (leaky-wave metasurface) antenna, beam scanning is fundamentally obtained by exciting and controlling specific Floquet modes of a periodic surface. That could be achieved through three steps of guided wave excitation, periodic modulation by the holographic Surface, and mode selection. The guided wave excitation is achieved by feed structures such as microstrip line, waveguide, or SIW that launches a guided wave with propagation constant *β* along the surface. That waves do not radiate. Consequently, periodic modulation by holographic surfaces such as slots, patches, vias, or meta-atoms that is engineered with periodic impedance modulation should be designed to create spatial harmonics (Floquet modes) of *β*_*n*_ *= β*_*o*_*+(2nπ/p).* This is the key mechanism of converting a bound guided wave into radiating Floquet modes by periodic modulation. One mode (usually *n* = − 1) leaks energy into free space at angle $\theta$. That mode is engineered to satisfy *β*_*n*_ $\approx$*K*_*o*_ *sin* $\theta$. The beam scanning is achieved by controlling which Floquet mode radiates and at specific angle. That could be achieved by frequency scanning (change operating frequency leads to change *k*_*o*_​ and effective *β*, $\theta =si{n^{ - 1}}\left( {\frac{{{\beta _n}}}{{{k_o}}}} \right)$*)*, or use active elements such as varactors, PIN diodes which modifies surface impedance then changes *β*, Shifts Floquet mode phase constant result in a continuous beam steering at fixed frequency or phase gradient (Holographic Control) based on holography. To control Floquet modes effectively period *p* determines angular spacing of modes Smaller *p* achieves wider scanning range, modulation depth Controls leakage r-ate (radiation efficiency). High modulation depth leads to strong radiation but short aperture, low modulation depth creates weak radiation. Propagation Constant *β* can be controlled via substrate, geometry, or tuning directly affects beam angle^50^. The periodic discontinuity that enables a part of the surface waves to leak out into the free space. The surface impedance distribution over the hologram structure determines the beam direction and is described by Eq. (3) where ${X_o}$ is the average amplitude of surface impedance, ${\Psi _{obj}}$ is the leaky waves field,${\mathrm{~}}{\Psi _{ref}}$ is the surface waves and *M* represents the modulation coefficient^50^:

3 $${z_s}\left( {x,y} \right)=j{X_o}\left[ {1+M \times Re({\Psi _{obj}} \times \Psi _{{ref}}^{{\mathrm{*}}}} \right]$$

In this work, 1-D hologram structure is designed. Therefore, the surface wave propagates in x-direction and the beam is tilted in the xz-plane. The simplified surface impedance *Zs* is given by Eq. (4). Where *P* is the period of the hologram cell (AIS) and the direction of radiation beam, $\theta$ is obtained by Eq. (5). At small values of *M*, the radiation angle $\theta$ is approximated to Eq. (6), where ${\eta _o}$ is the free space wave impedance (377 Ω)^38^.

4 $${z_s}\left( x \right)=j{X_o}[1+M \times cos\left( {2\pi x/p)} \right]$$

5 $$\theta =\arcsin ({k_s}/{k_o} - 2\pi /{k_o}p)$$

6 $$\theta \approx \arcsin (\sqrt {1+X_{o}^{2}/\eta _{o}^{2}} - 2\pi /{k_o}p)$$

**Fig. 3:**
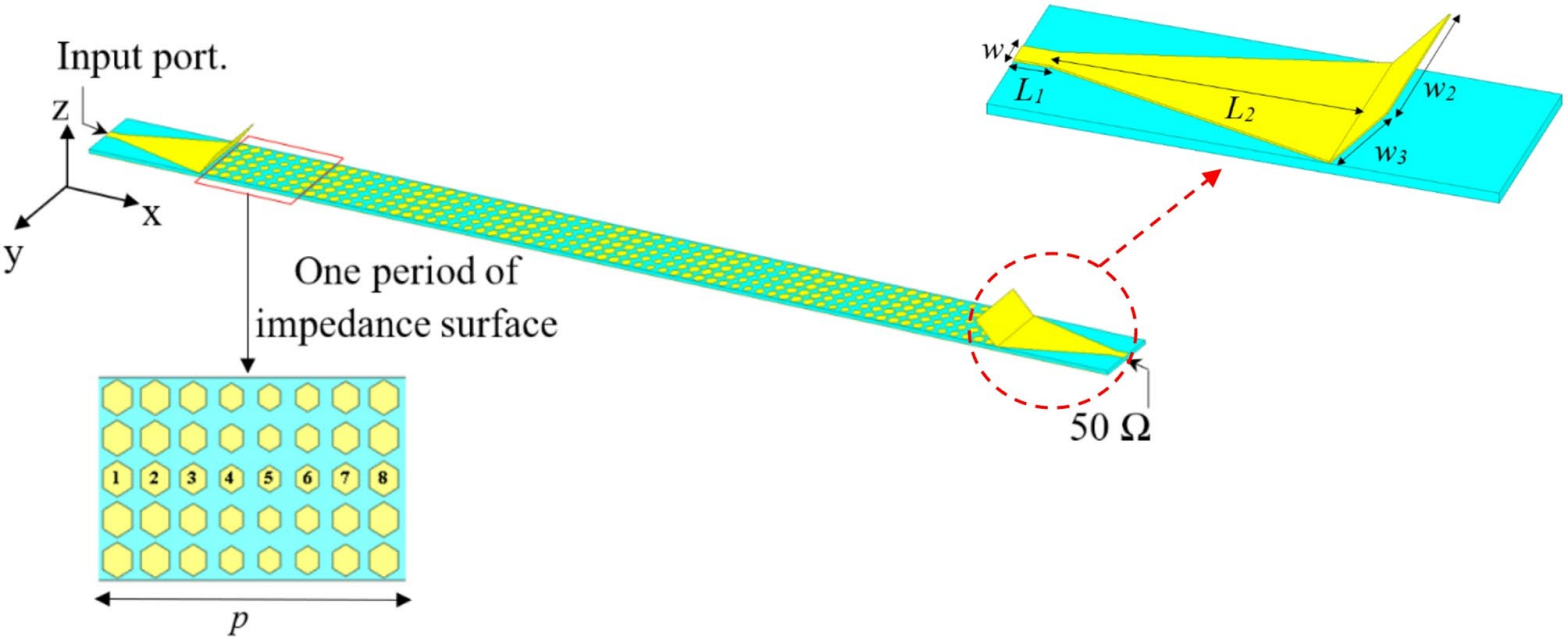
(a) Detailed structure of hologram antenna.

**Fig. 4:**
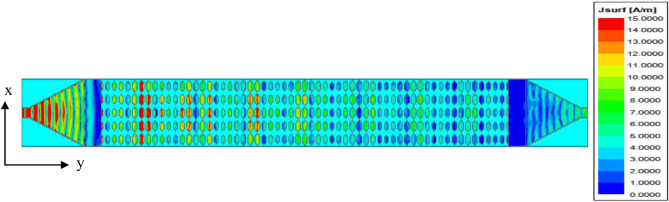
The current distribution at resonance frequency (16 GHz).

**Fig. 5:**
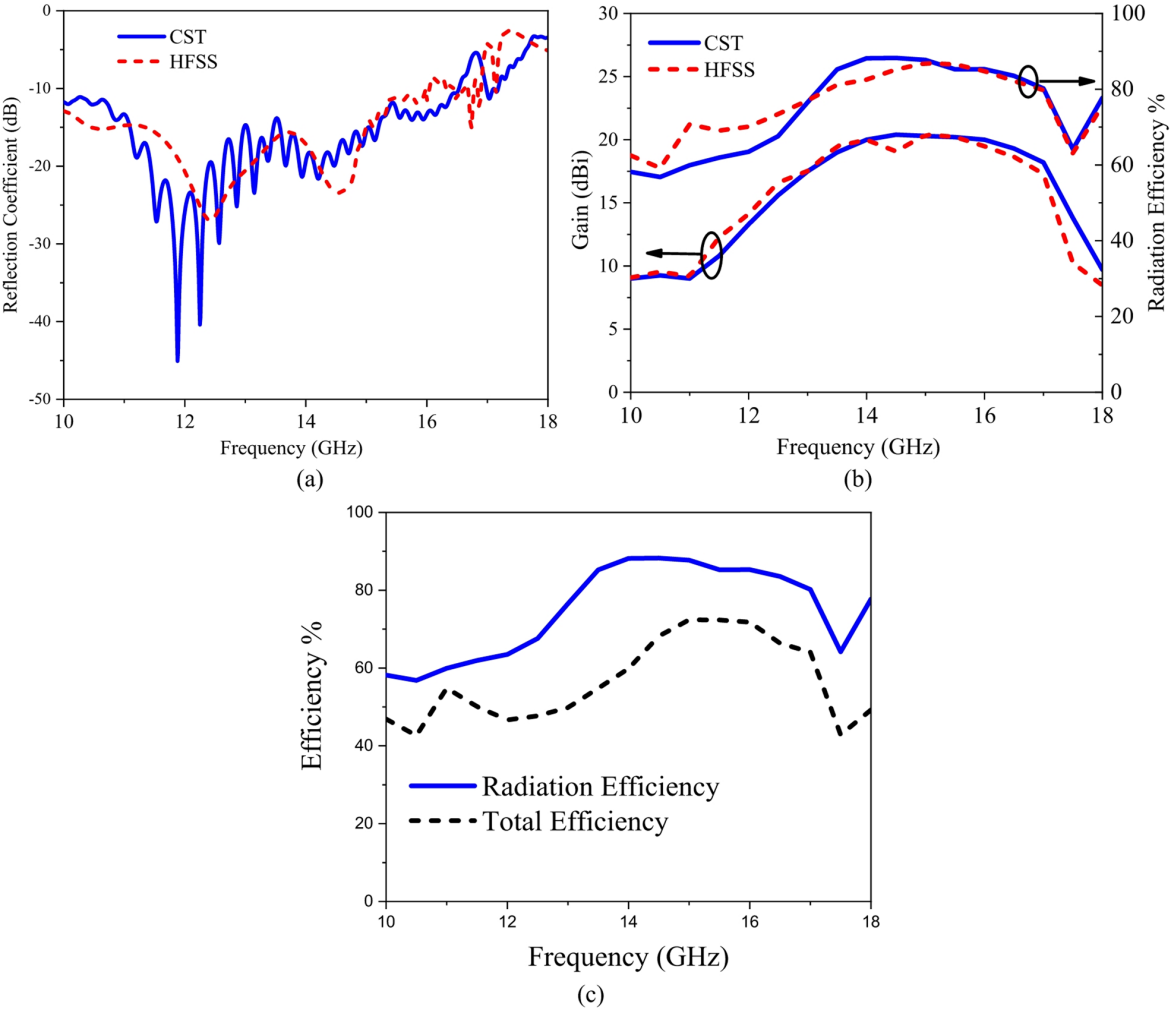
(a) Reflection coefficient, (b) Peak gain, the radiation efficiency, (c) radiation efficiency versus total efficiency variations with frequency for hologram antenna.

The configuration of proposed HA is indicated in Fig. 3. It consists of a periodical array from hexagonal patches. The antenna has a total area of 416 × 25 mm^2^ with one lattice period of impedance with length *p* = 40 mm. Two tapered microstrip transmission lines are used one (connected to the source) for exciting the antenna by generating surface wave and the other is connected to a 50 Ω terminal load. A slanted metal strip is placed with the tapered microstrip line for improving the impedance matching by avoiding electric field disorganizing in between surface wave launcher and metasurface^38^. Table 1 lists the dimensions of feeding. According to the modulated surface impedance, the hexagonal patches arranged along x-axis with different gap widths. Using Eq. (6), the lattice periodicity, *P*, for specific direction of radiated beam at certain frequency can be calculated. Here, the angle = 50° and 16 GHz is applied. The resultant periodicity of *P* is about 40 mm. The average value of surface impedance is *Xo* = 260 Ω, and the modulation coefficient is *M* = 0.2. To create the complete structure of proposed antenna, each period p of impendence surface is discretized equal number of cells *Nx* = 8 with spaced segments of *a* = 5 mm. The surface impedance, *Z*_*sn*_ corresponding to the gap width, *g*_*n*_, is calculated and listed in Table 2. Then, each period p (of eight-unit cells) was repeated five times rows to increase the antenna aperture. Moreover, six periods are repeated for increasing the antenna radiation efficiency. The current distribution at resonance frequency (16 GHz) is shown in Fig. 4. The designed hologram antenna creates a desired radiation pattern by modulating the surface current distribution, consequently it reconstructs a target electromagnetic wave (beam). Instead of feeding each element individually (like in phased arrays), the antenna uses a reference wave and modulation pattern to generate the required current. The current distribution on a hologram antenna surface is a modulated traveling surface current that is formed by interference between the reference wave and the desired radiation pattern. Which leads to reconstruct a beam in space. The meta surface is patterned with hexagonal patches and each patch scatters energy from the reference wave. The scattering strength, phase define the local current and form a coherent radiated beam. The current is not continuous, but sampled by the discrete hexagonal patch elements. Each element controls amplitude and phase. The current has traveling-wave nature, gradually leakage (power radiates as wave propagates), the phase gradient determines beam direction, and the amplitude taper controls sidelobes. The Current distribution is engineered, not natural, it strongly depends on feed location and surface impedance variation. The reflection coefficient variation versus frequency using full-wave simulations of HFSS and CST-MWS^49,51^ is shown in Fig. 5a. The antenna has good impedance matching with bandwidth spanning from 10 GHz to 16.5 GHz. Figure 5b indicates gain versus frequency with a peak value of 20.6 dBi and 1-dB bandwidth of 3 GHz with high efficiency of 87%. Radiation efficiency measures how effectively the antenna converts accepted power into radiated electromagnetic waves. It includes losses due to conductor resistance (ohmic losses), dielectric losses and ignores mismatch losses (reflection due to impedance mismatch). The radiation efficiency is given by Eq. (7), where ${R_{radiated}}$ is the radiation resistance, ${R_{loss}}$ is sum of conductor and dielectric loss resistances. Total efficiency measures how much of the input power from the source is actually radiated and is given by Eq. (8). It includes radiation efficiency losses and mismatch (reflection losses). The reflected power coefficient is ${\left| \Gamma \right|^2}$^52^.

7 $${\eta _{rad}}={p_{radiated}}/{p_{accepted}}=\frac{{{R_{radiated}}}}{{{R_{radiated}}+{R_{loss}}}}$$

8 $${\eta _{total}}={p_{radiated}}/{p_{input}}={\mathrm{~}}{\eta _{rad}} \times \left( {1 - {{\left| \Gamma \right|}^2}} \right)$$

**Fig. 6:**
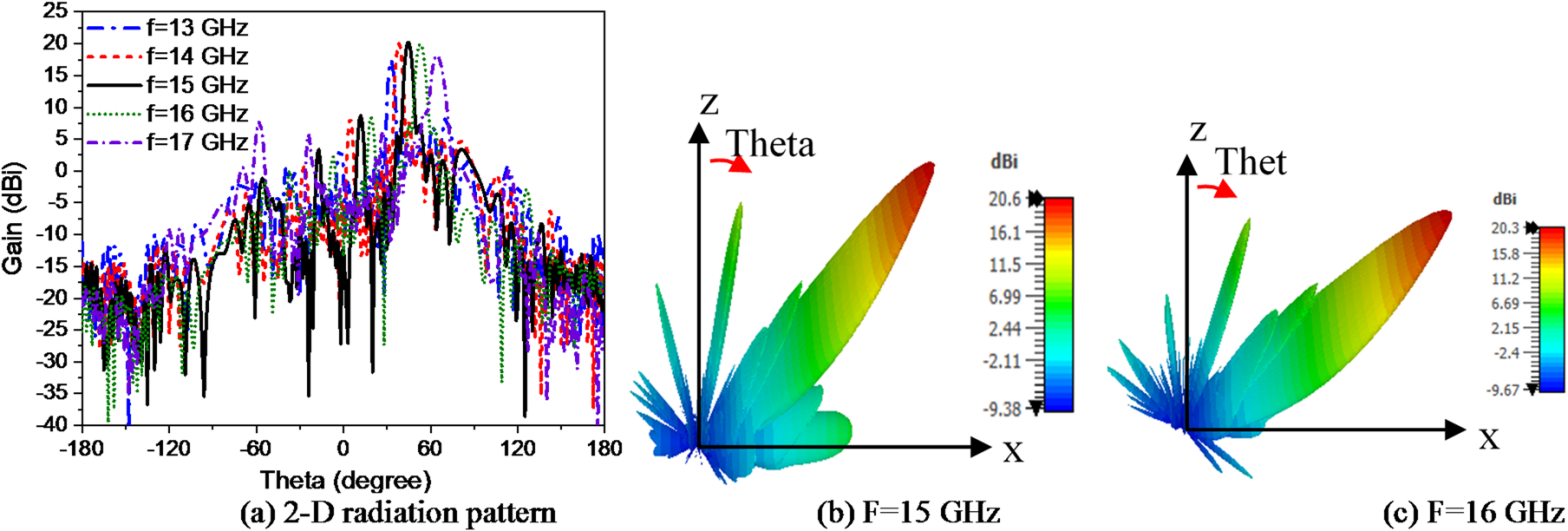
Hologram antenna (a) 2D radiation patterns at different frequencies, (b), (c) 3-D Radiation pattern at 15 GHz and 16 GHz.

The losses in copper and substrate for the proposed hologram antenna are considered during simulation and the efficiencies are given in Fig. 5c. The antenna has beam scanning with frequency as shown in Fig. 6(a). The direction of the radiated beam is changed according to the operating frequency. When the operating frequency is changed from 13 GHz to 17 GHz, the beam direction varies from 30° to 64°. The side-lobe level (SLL) is increased as the deflection angle is increased. The 3D radiation patterns of HA at 15 GHz, 16 GHz are shown in Fig. 6 (b) and (c) respectively.

**Table 1:** feed dimensions.

W_1_	W_2_	W3	L_1_	L_2_
3.5 mm	25 mm	15 mm	5 mm	40.5 mm

**Table 2:** Parameters of one lattice cell in one period of the proposed HMLWA Zs (Ω), g length (mm).

*N*_x_	1	2	3	4	5	6	7	8
*Z*_*s*_	339	323	283	243	226	243	283	323
*g*	1.1	1.2	1.5	1.9	2.1	1.9	1.5	1.2

## Dual and multi beam genus hologram antenna

**Fig. 7:**
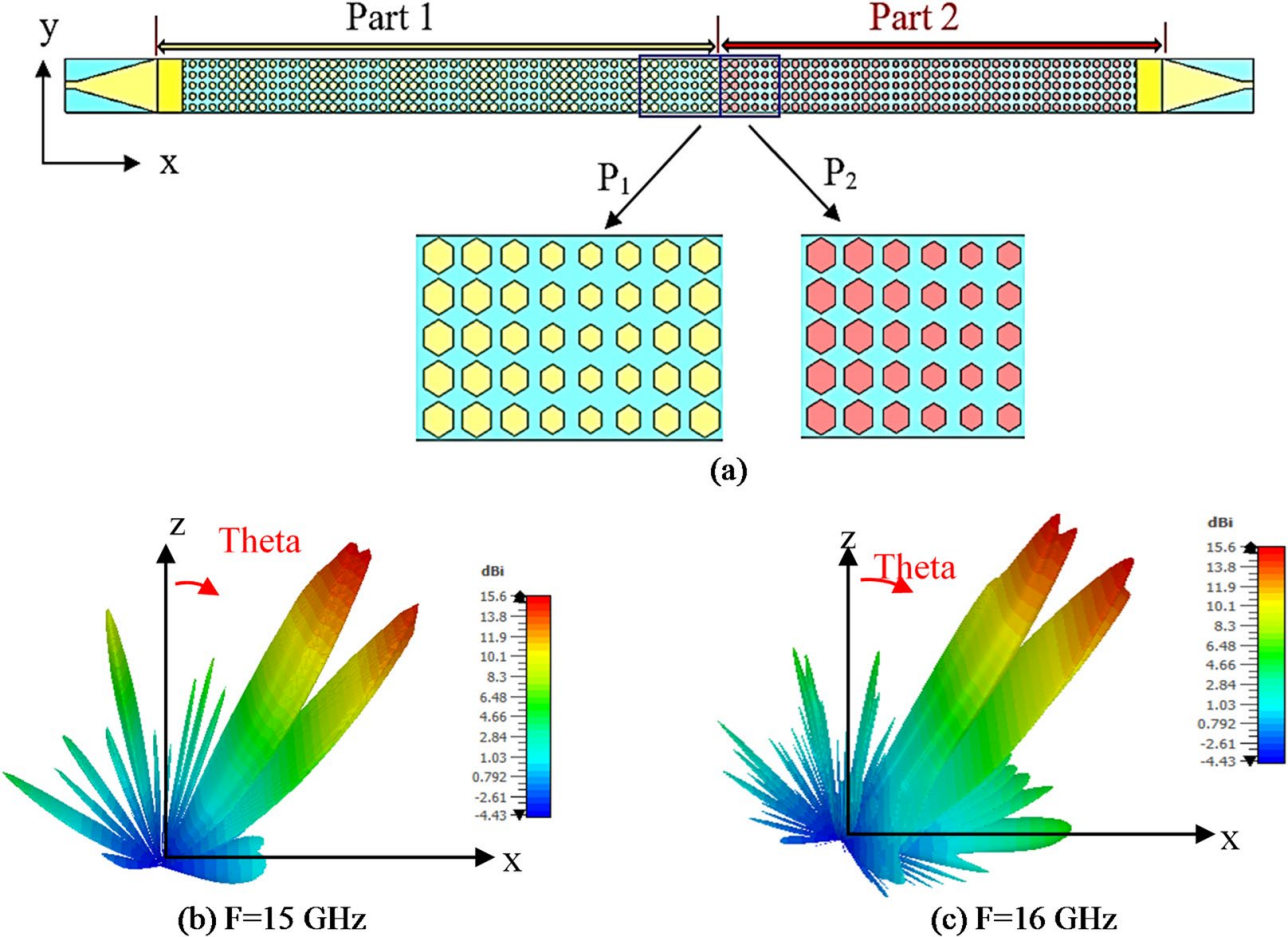
(a) Configuration of dual beam holographic antenna, (b), and (c) dual-beam antenna 3-D Radiation pattern at 15, 16 GHz.

**Fig. 8:**
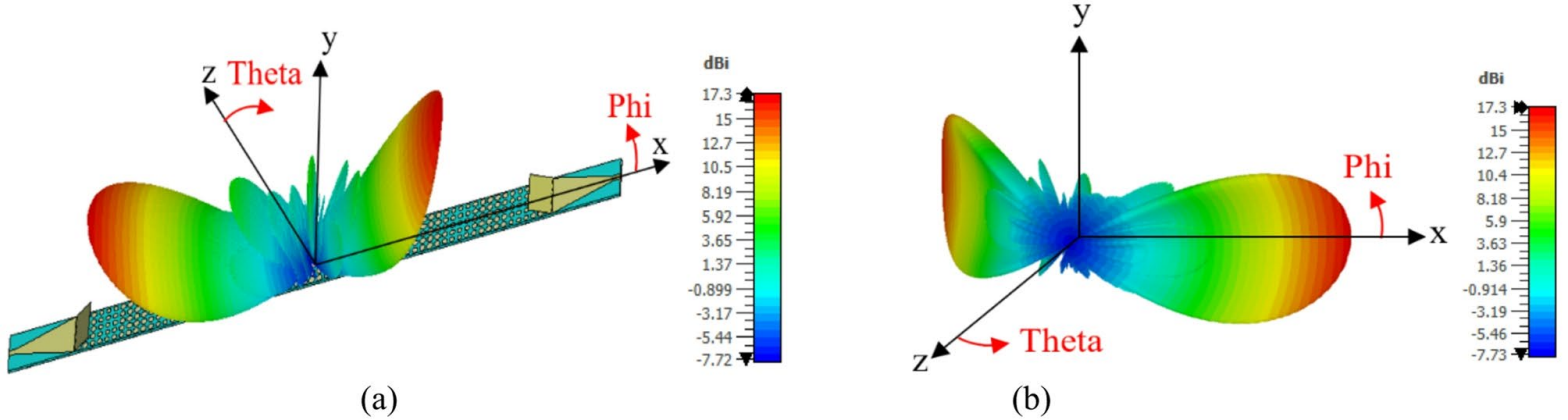
Antenna radiation patterns at 16 GHz (a) 60°, -60° and (b) at 120°, -120°.

**Fig. 9:**
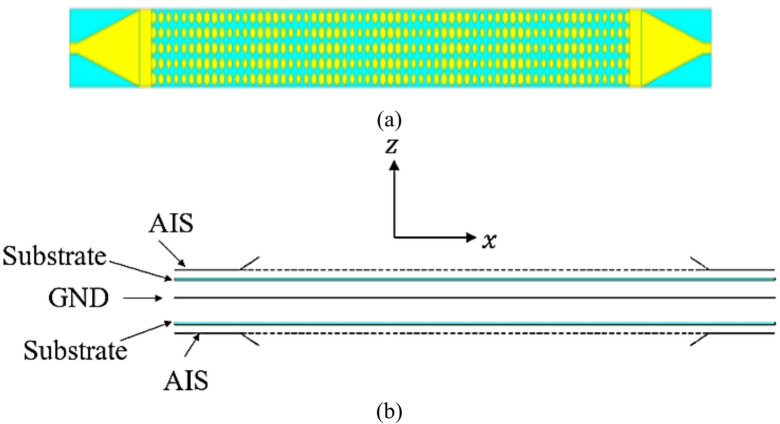
The structure of multi beam hologram antenna. (a) Top view, (b) side view.

**Fig. 10:**
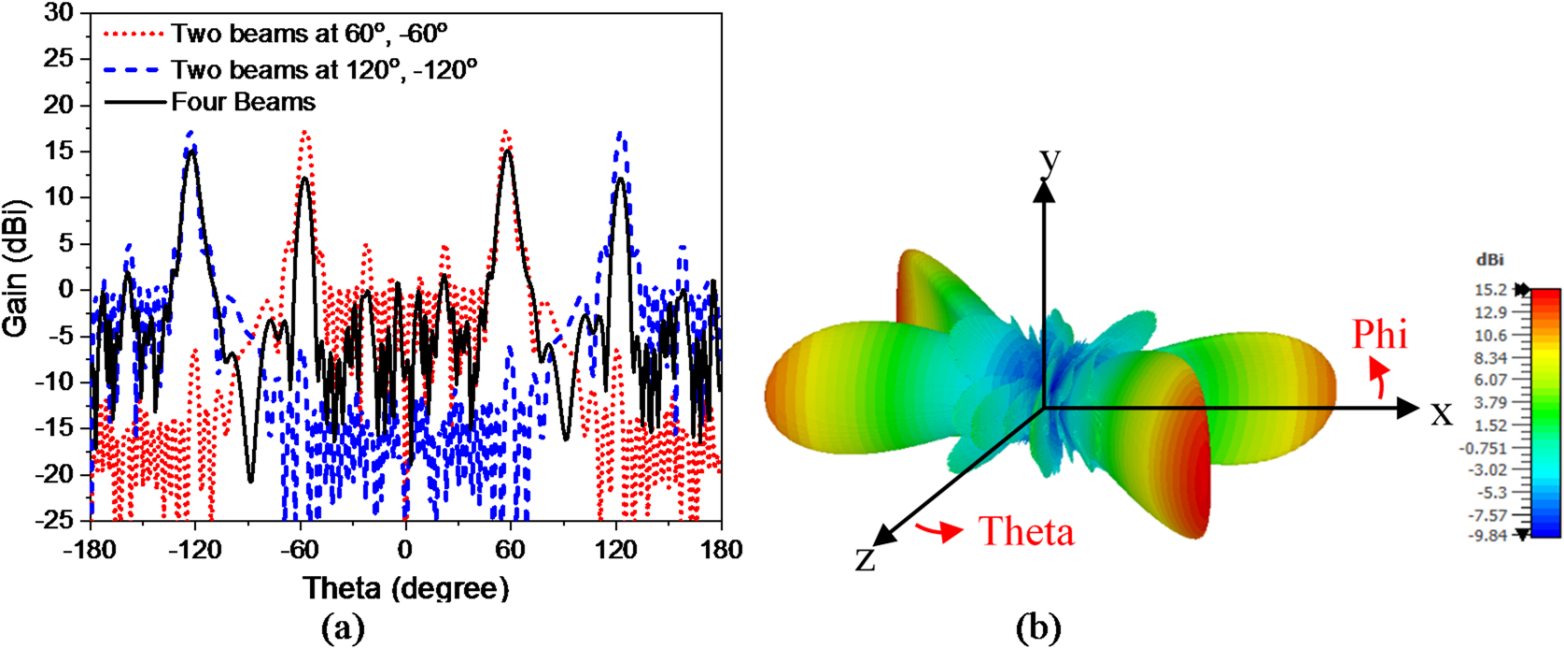
(a) 2-D radiation pattern of two beam antennas, four beam antenna, and (b) 3-D Radiation pattern for the multi beam antenna at 16 GHz.

According to Eq. (6), radiated beam is directed at different angles by changing the lattice periodicity. Depending on that, considering a structure consisting of two different lattice periodicities *P1* and *P2*, two different beams can be emitted from the antenna at angles ${\theta _1},{\mathrm{~}}and{\mathrm{~}}{\theta _2}$^53^. This is indicated in Fig. 7(a) which presents the structure of dual-beam antenna. Two different lattice periodicities of *P1* = 40 mm and *P2* = 30 mm is applied to radiate dual beam at ${\theta _1}=$30o, and ${\theta _2}=$40o. Eight cells are used for periodicity *P1*, and six cells are used for periodicity *P2*, because unit cell element has side length of 5 mm. Figures 7 (b), (c) show the dual beam radiation patterns at 15, and 16 GHz with gain of 15.6 and 15.9 dBi, respectively. The dual beams are scanned with changing frequency, where the dual beams directions are ${\theta _1}=$32°, ${\theta _2}=$42° at 15 GHz and $\theta _{1}^{{{\prime }}}=$29°, $\theta _{2}^{{{\prime }}}=$41° at 16 GHz. Two beams at 60°, -60° are obtained by changing the periodicity. Using periodicity of 50 mm, Table III shows the parameters of one lattice cell in one period 60° beam with *Nx* = 10. The antenna is fed simultaneously by two ports, one on the left and the other on the right which generates two beams at 60°, -60°. Using the same concept Two beams at 120°, -120° are achieved and Figs. 8 (a), (b) show the dual beam radiation patterns at 16 GHz. Antenna with four beams at 120°, 60°, -60°, -120° is designed using a two layers genus structure as shown in Fig. 9 (a) and (b). The top layer radiates beams at 60°, -60°, the bottom layer radiates beams at 120°, -120°, with a ground plane between two layers to ensure isolation and avoid interference. The thickness of the ground is 0.035 mm. Figure 10 (a) indicates comparison between 2-D radiation patterns of dual beam and four beam antennas at 16 GHz. The 3-D radiation pattern of four beam antenna at 16 GHz is presented in Fig. 10 (b) with maximum gain of 15.2 dBi.

**Table 3:** Parameters of one lattice cell in one period of dual beam hologram antenna at 60°, -60°, Z_s_ (Ω), g length (mm).

*N*_x_	1	2	3	4	5	6	7	8	9	10
*Z*_*s*_	313	235	231	311	240	227	311	244	234	310
*g*	1.35	2	2.05	1.35	1.95	1.6	1.35	1.9	2	1.35

## MIMO hologram antenna

**Fig. 11:**
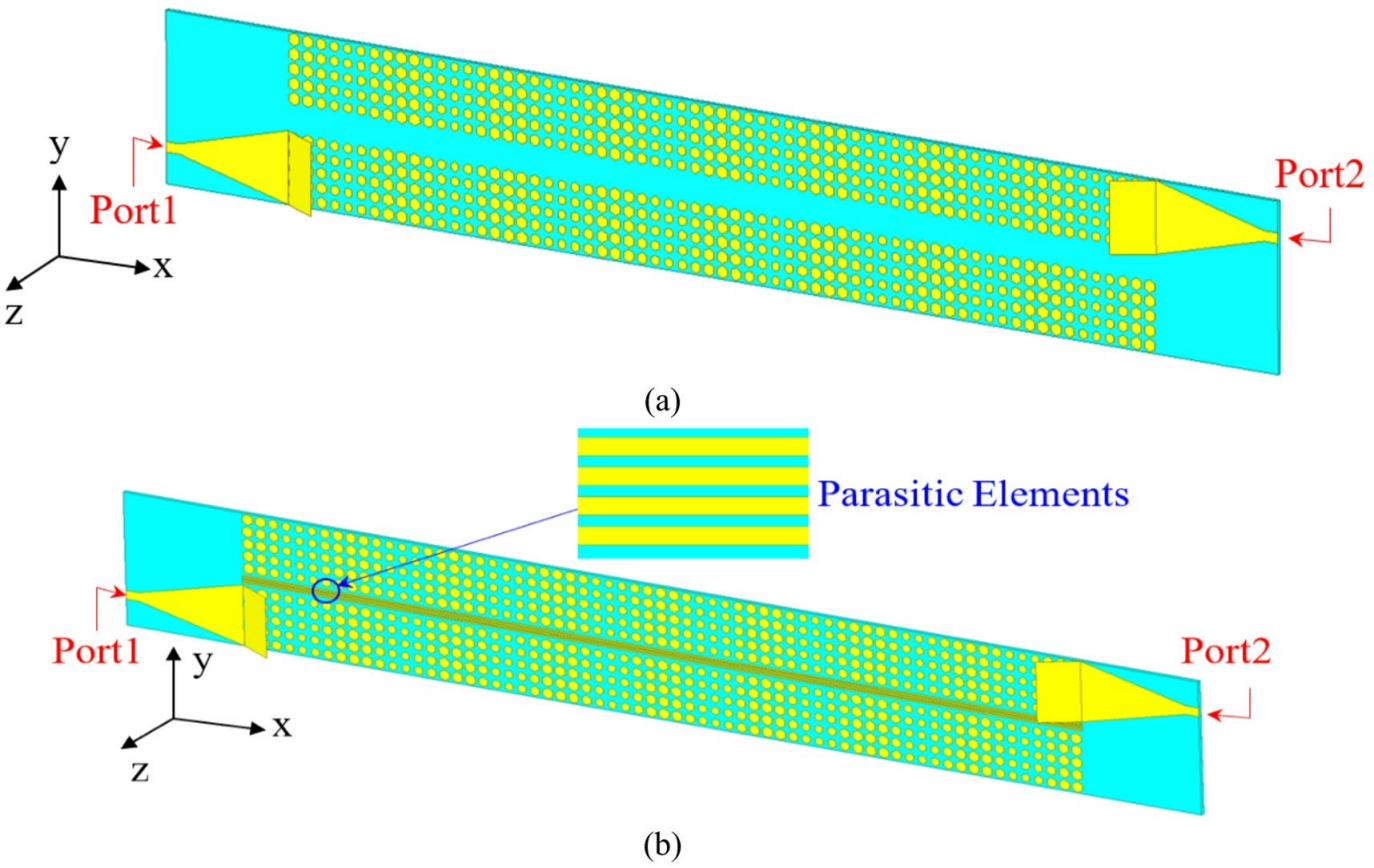
Structure of MIMO hologram antenna, (a) without parasitic elements (b) with parasitic elements.

**Fig. 12:**
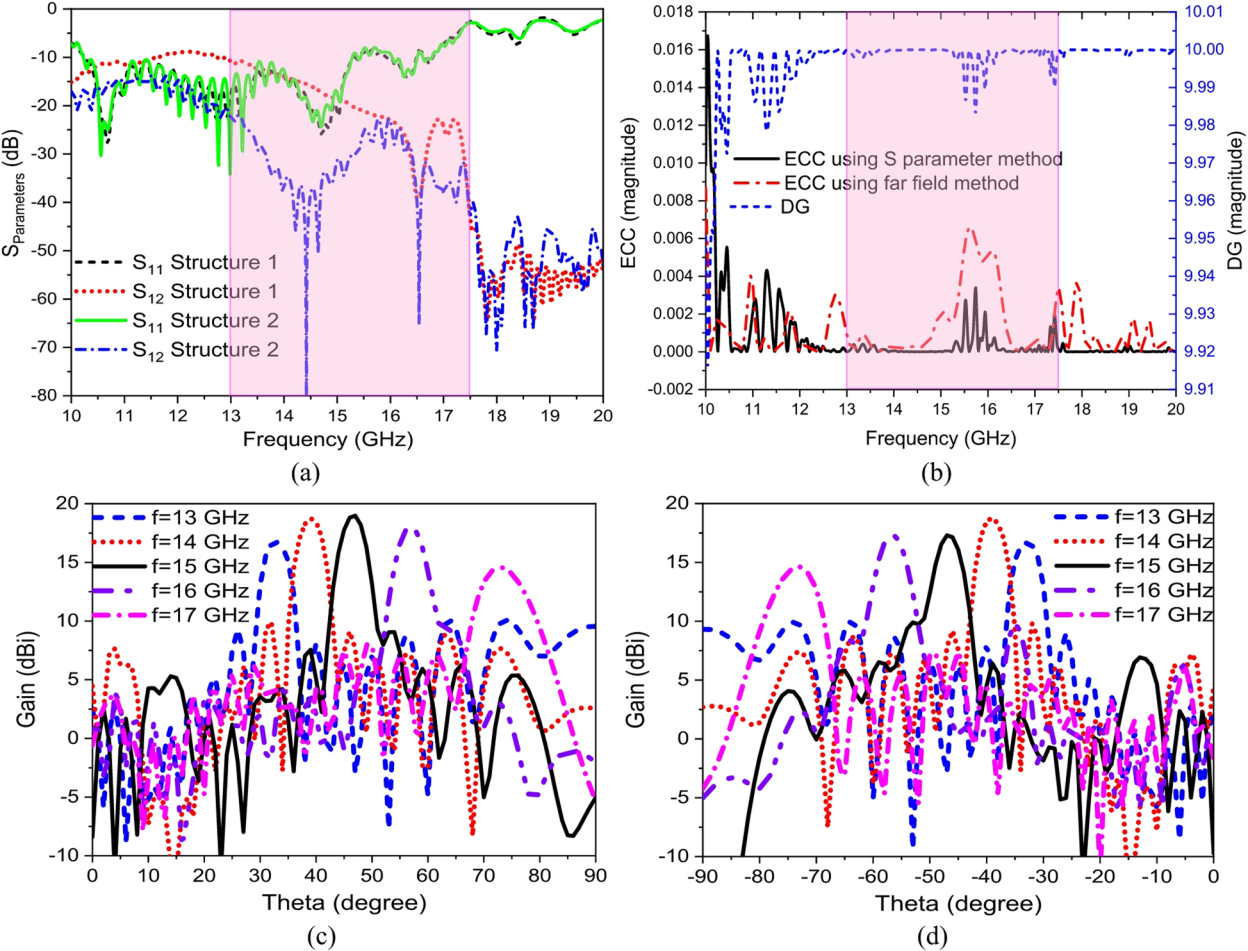
Hologram MIMO antenna (a) scattering parameters, (b) ECC and DG characteristics (c), (d) radiation patterns from port 1 and port 2, respectively.

Two hologram antenna elements are arranged in MIMO structure as shown in Fig. 11 (a). The MIMO antenna has total area of 416 × 54.7 mm^2^ with compact spacing between two antennas of 4.7 mm (${\lambda _o}/4$), where$~{\lambda _o}$ is the free space wave length at 16 GHz. To reduce the mutual coupling between the MIMO antenna elements, parasitic elements are inserted between the resonating antennas. These parasitic elements produce a coupling that counteracts the initial one induced by the excited antenna element. Consequently, the resulting coupled field will be in opposition to the passive antenna, resulting in the suppression of mutual coupling effects from one radiating patch to another^3^. The idea is to cancel or suppress the coupling fields between antenna 1 that radiates and induces undesired current in antenna 2. Consequently, parasitic elements are placed to support an induced current with opposite phase which prevents current from flowing toward adjacent antenna and leads to cancellation of coupling field. This achieves near-field shielding that blocks, redirects near fields. The parasitic elements act like a passive electromagnetic barrier and the coupling strength is reduced by minimizing the gap between parasitic elements. The four parasitic elements have length, width, separation of 325 mm, 0.55 mm, and 0.9 mm, respectively and are placed in the spacing between the two elements as shown in Fig. 11(b). Consequently, the mutual coupling is reduced and that can be seen in Fig. 12 (a) which indicates the effect of parasitic elements on the mutual coupling reduction. The amount of isolation and correlation between the line communications is the Envelope Correlation Coefficient (ECC). ECC is a basic parameter that estimates the diversity performances in MIMO antenna systems, it is important for approving the competency of the designed hologram MIMO antenna, and it should accomplish a very low ECC. The ECC is calculated using both S parameter method (Eq. 9)^54^ and far field method (Eq. 10)^55^ for better accuracy. Where ${F_1}$, ${F_2}~$are the fair field complex vectors of antenna 1 and 2, respectively, and dΩ =$\sin \theta d\theta d\varphi$ (Solid angle element). The Diversity Gain (DG) of the hologram MIMO antenna is also an important parameter to evaluate the antenna. The Diversity Gain can be calculated using Eq. 11. The proposed MIMO hologram antenna has low ECC due to the low mutual coupling between antenna elements which reflected into the calculation of ECC and high DG ~ 10. A low value of ECC was targeted to get better diversity gain, higher channel capacity, and improves signal reliability in multipath environments. Generally, in MIMO ECC < 0.05 gives excellent performance, ECC < 0.1 is good/ acceptable for most designs, and ECC < 0.3 is upper acceptable that is limited to some systems. Even if the MIMO antenna has good return loss and good isolation, low ECC is required to ensure real MIMO performance.

9 $$~~~~~~~~~~~~~~~~~~~~~~~~~~~~~~~~~~~~~~~~~~~~~ECC\left( {S~parameter~method} \right)=\frac{{\left| {S_{{11}}^{{\mathrm{*}}}{S_{12}}+S_{{21}}^{{\mathrm{*}}}{S_{22}}} \right|}}{{\left( {1 - {{\left| {{S_{11}}} \right|}^2} - {{\left| {{S_{21}}} \right|}^2}} \right)\left( {1 - {{\left| {{S_{22}}} \right|}^2} - {{\left| {{S_{12}}} \right|}^2}} \right)}}$$

10 $$ECC\left( {{\mathrm{far~field~method~}}} \right)=\frac{{{{\left| {\iint {F_1}\left( {\hat {r}} \right).F_{2}^{*}~\left( {\hat {r}} \right)d\Omega } \right|}^2}}}{{\left( {\iint {{\left| {{F_1}\left( {\hat {r}} \right)} \right|}^2}d\Omega } \right)\left( {\left| {{F_2}\left( {\hat {r}} \right)d\Omega } \right|} \right)}}$$

11 $$DG=10\sqrt {1 - {{\left( {ECC} \right)}^2}}$$

**Fig. 13:**
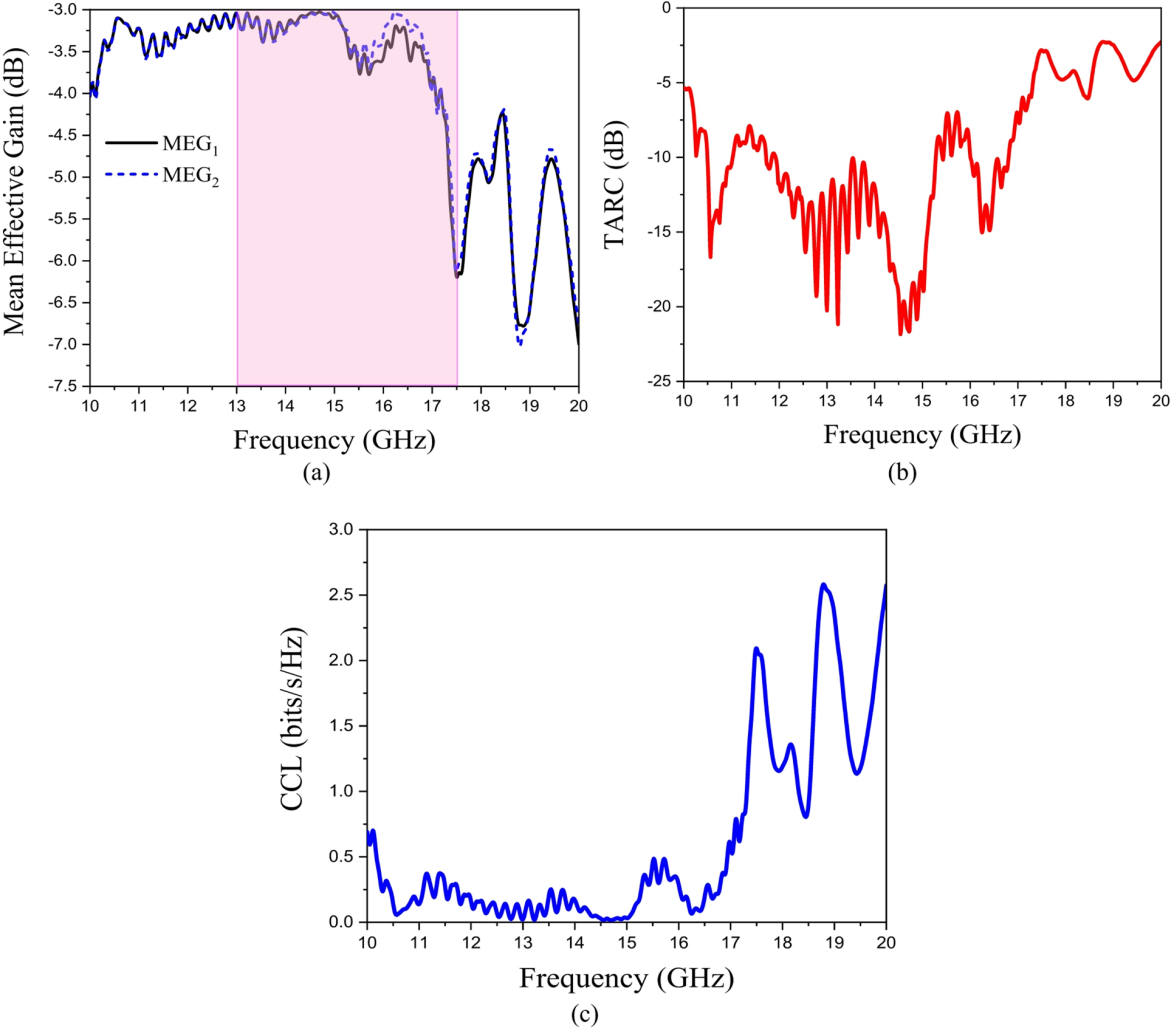
Hologram MIMO antenna (a) Mean effective gain, (b) Total Active reflection coefficient, and (c) Channel capacity loss variations versus frequency.

The MIMO acceptable values are ECC < 0.5 and DG ~ 10. The ECC and DG of the proposed hologram MIMO antenna is shown in Fig. 12(b). The proposed hologram MIMO antenna shows good diversity performance as the value of ECC is almost < 0.003 at the frequency band of 13–17.5 GHz. The value of DG is almost near to 10. The designed MIMO antenna has the ability of beam scanning at different frequencies with high gain value of 18.8 dBi at 14 GHz. Figures 12 (c), (d) show the 2-D radiation pattern of the designed MIMO antenna. The hologram MIMO antenna parameters MEG, TARC, CCL are calculated using Eqs. (12), (13), and (14)^56^. Figure 13 shows the mean effective gain, total Active reflection coefficient, and channel capacity loss variations versus frequency. The total Active Reflection Coefficient (TARC) represents the overall reflection coefficient when both ports are excited simultaneously

12 $$TARC=\sqrt {\frac{{{{\left| {{S_{11}}+{S_{21}}{{\mathrm{e}}^{j\theta }}} \right|}^2}+{{\left| {{S_{21}}+{S_{22}}{{\mathrm{e}}^{j\theta }}} \right|}^2}}}{2}}$$

where θ is the phase difference between the two excitation signals (usually swept from 0° to 180°). Acceptable value TARC < -10 dB across the band is designed. The Mean Effective Gain (MEG) is

13 $$ME{G_i}=0.5\left( {1 - \mathop \sum \limits_{{j=1}}^{N} {{\left| {{S_{ij}}} \right|}^2}} \right){\mathrm{~~~~~~~~~~~~~~~~}}\left( {for{\mathrm{~}}i=1,{\mathrm{~}}2} \right)$$

For good MIMO performance: |MEG_1_ - MEG_2_| < 3 dB. The Channel Capacity Loss (CCL)

14 $$CCL= - lo{g_2}~~det\left( X \right)~~$$

Where $$X=\left[ {\begin{array}{*{20}{c}} {1 - {{\left| {{S_{11}}} \right|}^2} - {{\left| {{S_{21}}} \right|}^2}~~~~~~~~~~}&{ - \left( {S_{{11}}^{{\mathrm{*}}}{S_{12}}+S_{{21}}^{{\mathrm{*}}}{S_{22}}} \right)} \\ { - \left( {S_{{12}}^{{\mathrm{*}}}{S_{11}}+S_{{22}}^{{\mathrm{*}}}{S_{21}}} \right)}&{1 - {{\left| {{S_{22}}} \right|}^2} - {{\left| {{S_{12}}} \right|}^2}} \end{array}} \right]~~~~~~$$and the acceptable value is CCL < 0.4 bits/s/Hz.

## Conformal hologram antenna

**Fig. 14:**
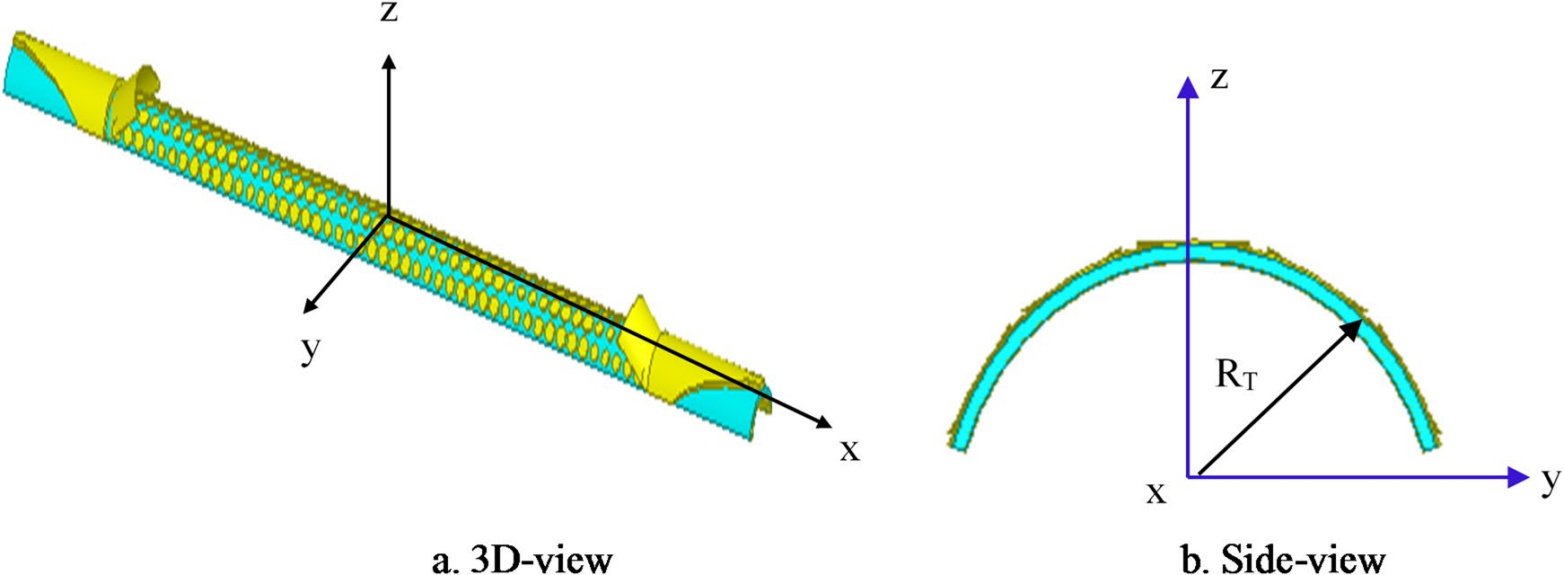
The structure of conformal hologram antenna along its transverse direction (around y-axis) direction.

**Fig. 15:**
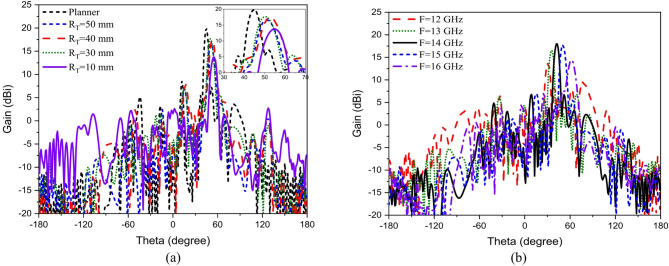
Radiation pattern of conformal (transverse direction) HA (a) with different radii of curvature at f = 15 GHz, (b) at different frequencies with radii of curvature R_T_ = 30 mm.

**Fig. 16:**
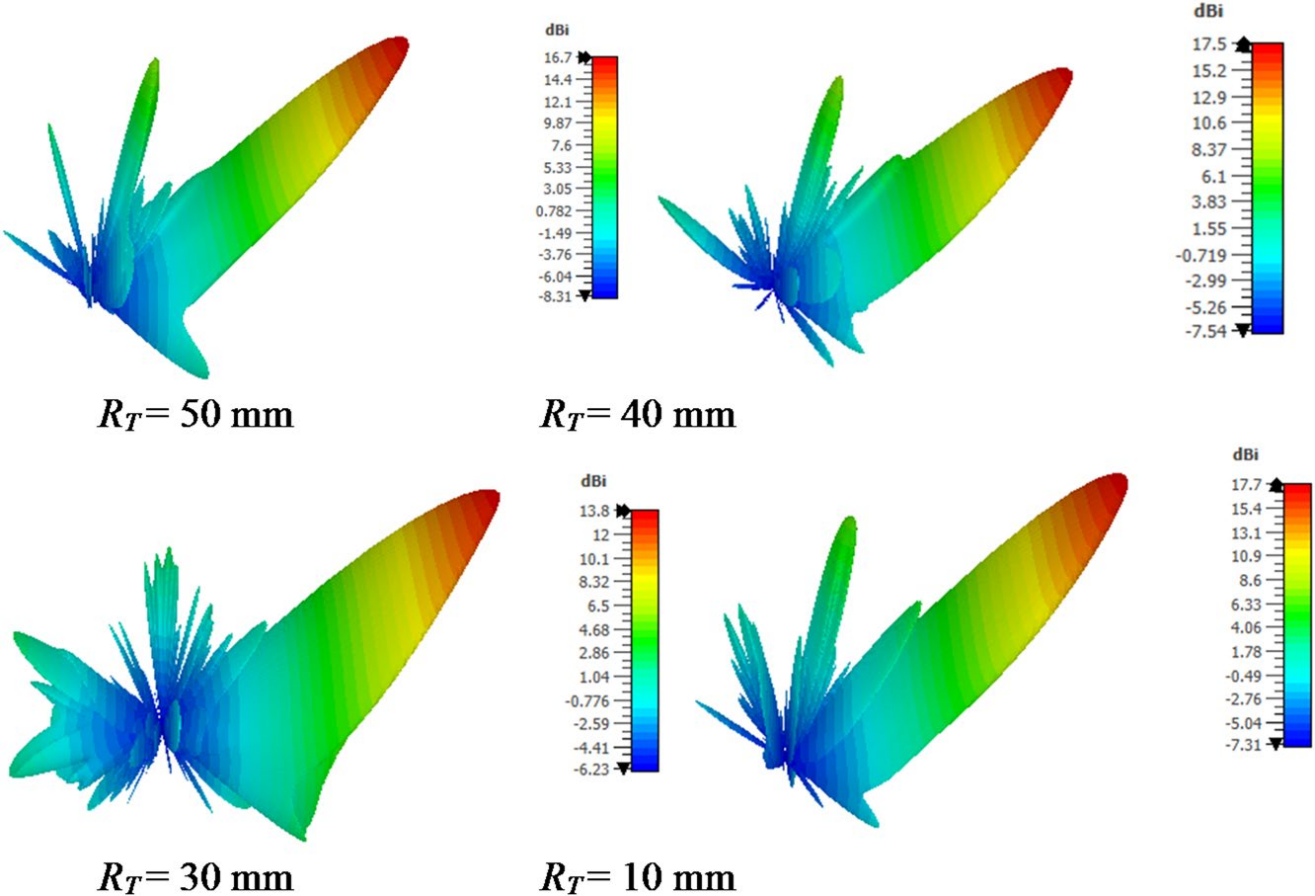
A 3-D radiation patterns of curved hologram antenna with different radius *R*_*T*_ along its transverse direction at 15 GHz.

**Fig. 17:**
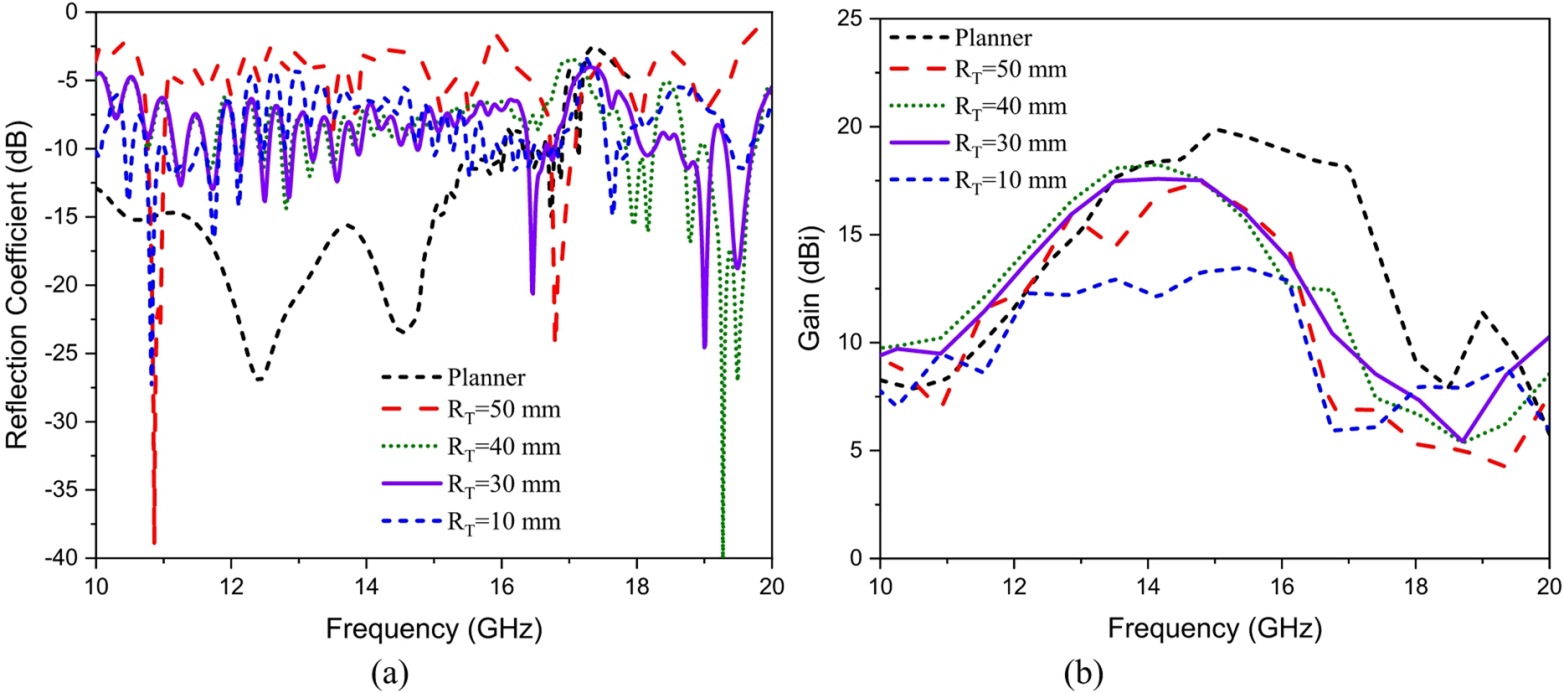
(a) Reflection coefficient, (b) Gain variation versus frequency for conformal hologram antenna (transverse direction) at different radii of curvature.

**Fig. 18:**
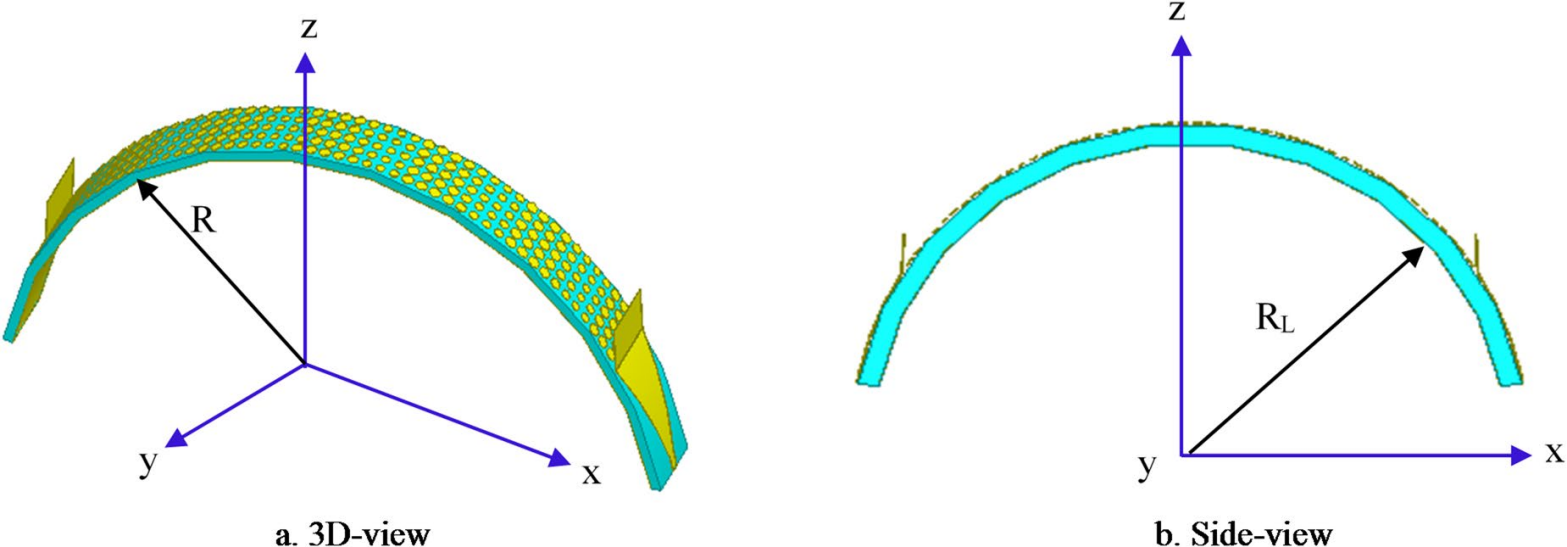
Structure of conformal HA along its longitudinal direction (around x-axis).

**Fig. 19:**
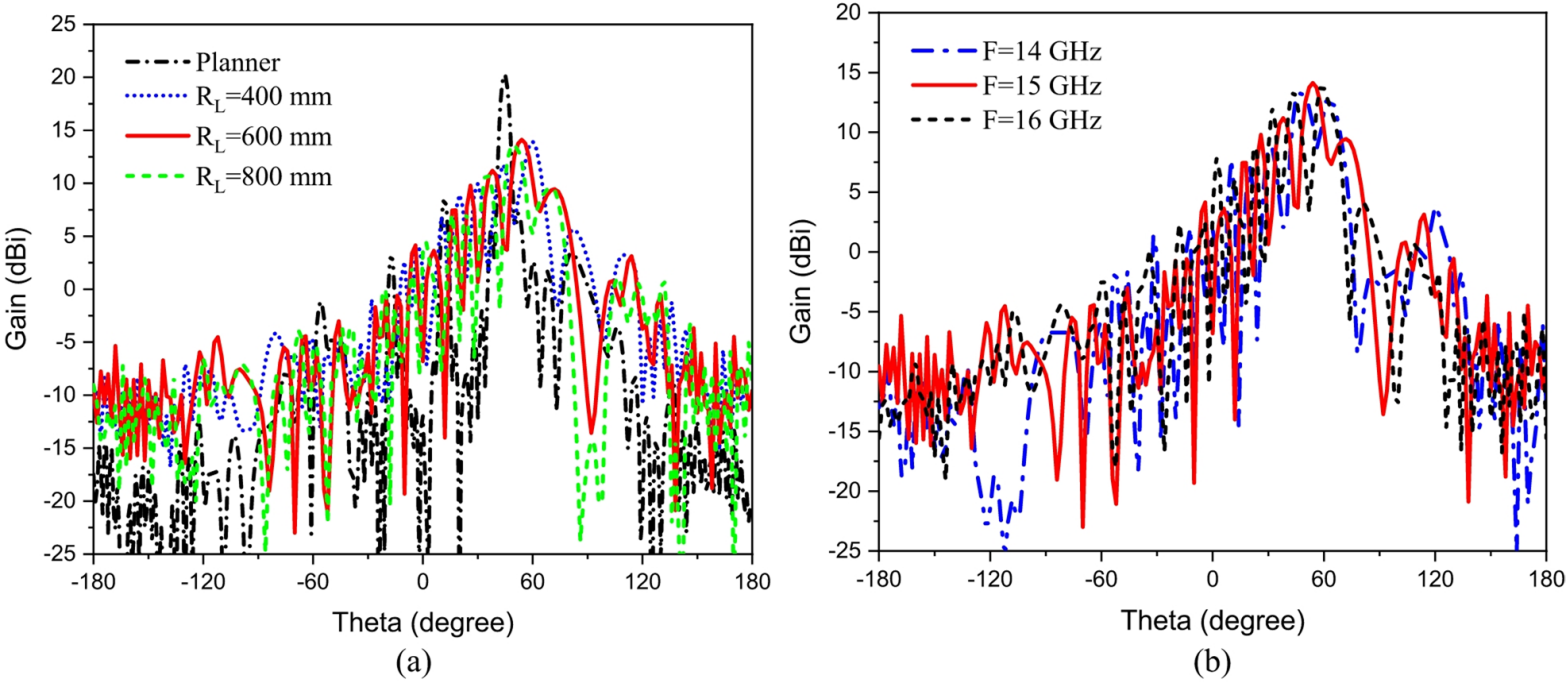
Conformal (longitudinal direction) hologram antenna (a) Radiation pattern with different radii of curvature at *f* = 15 GHz, (b) Radiation pattern at different frequencies with radii of curvature *RL* = 600 mm.

**Fig. 20:**
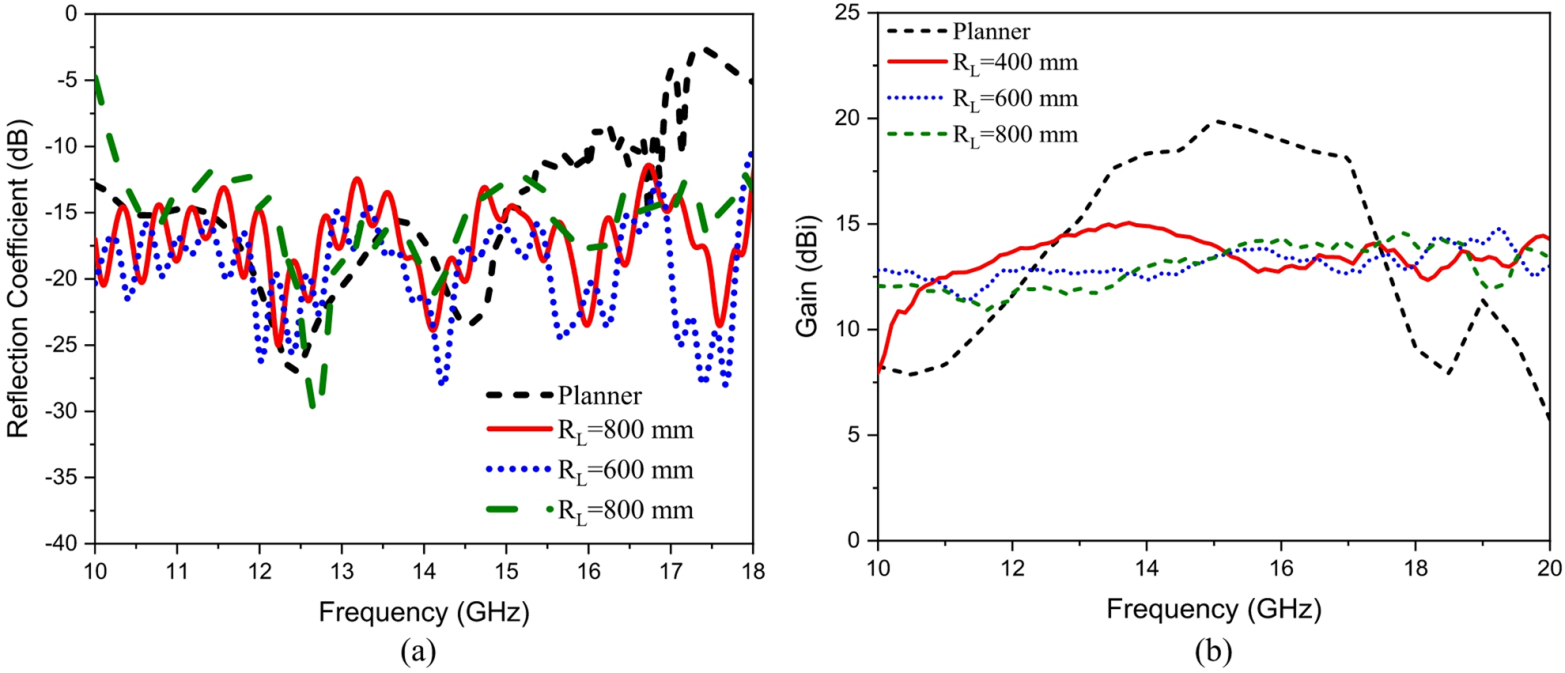
(a) Reflection coefficient and (b) gain variation versus frequency at different radii of curvature.

Conformal HA is proposed due to its essential requirement in different applications that require integration of an antenna radiator on conformal surfaces, such as missiles, cars, trains, aircraft^57^. The proposed conformal HA is studied in two cases of curvatures, transverse direction case (around y-axis) and longitudinal direction case (around x-axis). The conformal HA in transverse direction case (around y-axis) is shown in Fig. 14. The hexagonal patches periodic array and the two tapered microstrip lines are curved to follow surface curvatures. Different radii of curvatures are investigated and corresponding gain patterns at 15 GHz are shown in Fig. 15 (a). The results indicate that the peak gain is changed with changing the radii of curvature. The maximum value of gain is 17.7 dBi with *R*_*T*_ = 30 mm and the minimum gain is 13.8 dBi with *R*_*T*_ = 10 mm at 15 GHz. Furthermore, the conformal antenna in transverse direction has the ability of beam scanning at different frequencies with constant value of curvature radii that as indicated in Fig. 15 (b). Figure 16 shows the 3-D radiation patterns of antenna with different values of radius *R*_*T*_ along its transverse direction at 15 GHz. The reflection coefficient variation versus frequency for conformal HA in the transverse direction with different radii of longitudinal curvatures *R*_*T*_ is indicated in Fig. 17 (a). The gain variation versus frequency for antenna at different radius of curvature is shown in Fig. 17(b). It indicates that the frequency corresponding to the maximum gain is decreased with increasing the curvature of the antenna. When the curvature of the antenna increases, the operating frequency is varied, and the peak gain is decreased. The conformal HA in the longitudinal direction case (around x-axis) is shown in Fig. 18. Different radii of longitudinal curvatures *R*_*L*_= 4 cm, 6 cm, and 8 cm are investigated and the corresponding gain patterns at 15 GHz are shown in Fig. 19 (a). With decreasing the radii of curvature, the peak gain is decreased, and the side lobe level is increased. Compared to the planar structure and the curved along transverse direction, the curved antenna along the longitudinal direction effects the half power beam width (HPBW) of the radiation pattern. Getting more wider HPBW in the case of longitudinal direction than HPBWs in the planar antenna and curved antenna along transverse direction. That can be observed in Fig. 15 (a) and Fig. 19 (a). The curved antenna in longitudinal direction has the ability of beam scanning at different frequencies. The radiation patterns at different frequencies with radii of curvature *RL* = 6 cm is shown in Fig. 19 (b). The reflection coefficient variation versus frequency for conformal HA in the longitudinal direction with different radii of longitudinal curvatures *R*_*L*_= 4 cm, 6 cm, and 8 cm is indicated in Fig. 20 (a). Figure 20 (b) shows the gain variation versus frequency for curved antenna in longitudinal direction.

The above study of the two cases of curvatures, the transverse direction and longitudinal direction, shows that the curvature of proposed antenna in transverse direction has less effect on the antenna parameters than in longitudinal direction. This because that the proposed antenna is a leaky wave antenna which it’s radiating aperture is larger in the longitudinal direction than in the transverse direction. The travelling wave radiates a fan-shaped beam, which is tilted and more directive in the longitudinal direction, compared to the shorter transverse direction. Therefore, the effect of the curvature is more evident when this antenna is conformed along its longitudinal plane where the beam is narrowed and scanned.

Table 4 indicates comparison between the proposed hologram antenna and related papers in the literature. It can be noticed that the proposed hologram antenna has wide bandwidth, wide scanning rang, high gain, and multi beams.

**Table 4:** Comparison of proposed hologram antenna with papers in literature.

Ref.	F_o_ (GHz)	Bandwidth (GHz)	Aperture area	Peak gain (dBi)	Scanning range	Number of beams
^58^	9	8.6–10	18.3 × 0.8 *λ*_*g*_^*2*^	12	20°	One
^59^	10	-	24.9 *×* 0.25 *λ*_*g*_^*2*^	9	10°	
^60^	18	-	11.3 *×* 0.65 *λ*_*g*_^*2*^	11.3	20°	
^61^	9.3	8–11	15.9 *×*0.31 *λ*_*g*_^*2*^	12.2	20°	One
^62^	18	17–19	10.72* × *2.67 *λ*_*g*_^*2*^	19.7	18°	One
^38^	16	13-17.5	20.6* × *2.45 *λ*_*g*_^*2*^	17	39°	One
^63^	28	26–30	0.5*π* × (9.4*λ*_0_)^2^ = 138.8 *λ*_0_^2^	18.4	-	One
					Beam1(*θ*_*1*_ = 20°, *ϕ*_*1*_ = 45°) Beam2(*θ*_*2*_ = 50°, *ϕ*_*2*_ = 135°)	Two
					Beam1(*θ*_*1*_ = 15°, *ϕ*_*1*_ = 150°) Beam2(*θ*_*2*_ = 5°, *ϕ*_*2*_ = 60°) Beam3(*θ*_*3*_ = 45°, *ϕ*_*3*_ = 120◦) Beam4(*θ4* = 55, *ϕ*_*4*_ = 30◦)	Four
This work	16	10–16	25.2 *×*1.5 *λ*_*g*_^*2*^	20.6	34° (from 30 ° to 64 °)	One
					Beam1(${\theta _1}=$32°, ${\varphi _1}={90^o}$) Beam2(${\theta _2}=$42°, ${\varphi _2}={90^o}$)	Two
					Beam1(${\theta _1}=$29°, ${\varphi _1}={90^o}$) Beam2(${\theta _2}=$41°, ${\varphi _2}={90^o}$)	Two
					Beam_1_(${\theta _1}$= 120°, ${\varphi _1}={90^o}$) Beam_2_(${\theta _2}$ = 60°, ${\varphi _2}={90^o}$) Beam_3_(${\theta _3}$ = -60°, ${\varphi _3}={90^o}$) Beam_4_(${\theta _4}$ = - 120°${\varphi _4}={90^o}$)	Four

## Conclusion

This paper presents a novel Genus Hologram Antenna (GHA) based on surface impedance modulation using a periodic array of hexagonal meta-patches. The proposed antenna successfully integrates several key performance features high gain, wide-angle frequency beam scanning, multi-beam generation, compact MIMO operation, and conformal capability in a simple, low-cost, and low-profile single-layer structure without requiring complex feeding networks or phase shifters. The designed GHA achieves a wide beam-scanning range from 30° to 64° over the frequency band of 13–17 GHz, with a maximum realized gain of 20.6 dBi and radiation efficiency of 87%. By controlling the lattice periodicity, dual-beam radiation at different angle pairs and four simultaneous beams at (120°, 60°, − 60°, and − 120°) with a gain of 15.2 dBi have been realized. For MIMO applications, two GHA elements were integrated with an ultra-compact spacing of only 4.7 mm (0.25*λ₀* at 16 GHz). The strategic insertion of parasitic decoupling elements reduced the mutual coupling from − 10 dB to better than − 20 dB across the operating band, while maintaining excellent diversity performance (ECC < 0.003 and DG ≈ 10). Furthermore, the conformal behavior of the proposed antenna was thoroughly investigated under both transverse and longitudinal bending conditions. The study showed that bending the HA around the longitudinal direction has a higher effect on the antenna radiation parameters than bending it around the transverse direction. The results confirm that the antenna maintains acceptable performance when conformed on curved surfaces, making it highly suitable for integration on modern platforms such as missiles, unmanned aerial vehicles, automobiles, and high-speed trains. The proposed Genus Hologram Antenna offers a compelling solution that combines high electromagnetic performance with structural flexibility and practical implementation advantages. The GHA is suitable for next-generation 5G/6G wireless communication systems and multifunctional conformal platforms. The introduced hologram antenna can be modified by using reconfigurable material (such as plasma, graphene) or varactor diodes in the antenna structure. Consequently, the antenna will be reconfigurable which will improve the beam steering and change the antenna radiation pattern direction. In addition to that, two-dimensional hologram antenna can be implemented rather than the one-dimensional hologram. The two-dimensional hologram antenna will improve the antenna gain and directivity.

## Data Availability

The datasets used and/or analysed during the current study available from the corresponding author on reasonable request.
